# Pregnancy-associated breast cancer: nationwide Dutch study confirms a discriminatory aggressive histopathologic profile

**DOI:** 10.1007/s10549-021-06130-w

**Published:** 2021-02-26

**Authors:** B. B. M. Suelmann, C. van Dooijeweert, E. van der Wall, S. Linn, P. J. van Diest

**Affiliations:** 1 Department of Medical Oncology, University Medical Center Utrecht, PO Box 85500, Utrecht, GA 3508 The Netherlands; 2 Department of Pathology, University Medical Center Utrecht, Utrecht, The Netherlands; 3 Department of Medical Oncology, Netherlands Cancer Institute (AVL-NKI), Amsterdam, The Netherlands

**Keywords:** Pregnancy-associated breast cancer, Histopathology, Pregnancy, Lactation, Breast cancer

## Abstract

**Purpose:**

Breast cancer is the most common type of malignancy in pregnant women, occurring approximately once in every 3000 pregnancies. Pregnancy-associated breast cancer (PABC) is commonly defined as breast cancer diagnosed during or within one year after pregnancy, and it accounts for up to 6.9% of all breast cancers in women younger than 45 years old. Whether these cancers arise before or during pregnancy, and whether they are stimulated by the high hormonal environment of pregnancy, is currently unknown. This study assesses the histopathological profile of PABC in a large Dutch population-based cohort.

**Methods:**

We identified 744 patients with PABC (in this cohort defined as breast cancer diagnosed during or within 6 months after pregnancy) diagnosed between 1988 and 2019, in the nationwide Dutch Pathology Registry (PALGA). An age-matched PALGA cohort of unselected breast cancer patients (≤ 45 years), diagnosed between 2013 and 2016, was used as a control. Histopathologic features of both cohorts were compared.

**Results:**

The median age of PABC patients was 34.3 years old (range 19–45 years) and most breast cancers were diagnosed during pregnancy (74.2%). As compared to age-matched controls, PABC patients had tumors of higher Bloom–Richardson grade (grade I: 1.5% vs. 12.4%, grade II: 16.9% vs. 31.3%, grade III: 80.3% vs. 39.5%, *p* < 0.0001). Furthermore, estrogen (ER)- and progesterone (PR)-receptor expression was less frequently reported positive (ER: 38.9% vs. 68.2% and PR: 33.9% vs. 59.0%, *p* < 0.0001), while a higher percentage of PABC tumors overexpressed HER2 (20.0% vs. 10.0%, *p* < 0.0001). The most observed intrinsic subtype in PABC was triple-negative breast cancer (38.3% vs. 22.0%, *p* < 0.0001), whereas hormone-driven cancers were significantly less diagnosed (37.9% vs. 67.3%, *p* < 0.0001).

**Conclusion:**

This study, based on a large population-based cohort of 744 PABC Dutch patients, underlines the more aggressive histopathologic profile compared to age-matched breast cancer patients ≤ 45 years. Further in-depth genetic analysis will be performed to unravel the origin of this discriminating phenotype. It definitely calls for timely detection and optimal treatment of this small but delicate subgroup of breast cancer patients.

**Supplementary Information:**

The online version of this article (10.1007/s10549-021-06130-w) contains supplementary material, which is available to authorized users.

## Introduction

Breast cancer occurs in approximately one in every 3000 pregnancies [1–4], which makes this the most common type of malignancy in pregnant women [5]. Pregnancy-associated breast cancer (PABC) is commonly defined as breast cancer diagnosed during or within 1 year after pregnancy [6], and it accounts for up to 6.9% of all breast cancers in women younger than 45 years of age [7–9]. In women below the age of 35, the proportion of PABC even rises up to 15.6% [10]. The incidence of PABC has increased markedly during the last decades [7], and is expected to rise further, particularly in developed countries, due to the increasing age of (first) childbearing and an ongoing increase of young-onset breast cancer [5, 11, 12].

PABC is generally recognized as a particularly aggressive type of cancer for several reasons: occurrence in a younger population, an advanced T stage at diagnosis [10, 13–16], a high rate of lymph node involvement [10, 15], higher grade tumors [15], a negative estrogen (ER)- and progesterone (PR)-receptor status [13–15], and a higher rate of HER2 overexpression [16–21]. These characteristics, usually common in breast cancer at young age, are however described to differ in percentages of occurrence in PABC. In addition, gestational physiologic alterations in the breast commonly result in a delayed diagnosis of breast cancer and thereby more advanced stages [22]. Furthermore, whether these cancers arise before or during pregnancy, and whether they are stimulated by the high hormonal environment of pregnancy, is currently unknown.

The lack of a comprehensive understanding of the interaction between pregnancy and breast carcinogenesis indicates the need for more insights in the development of PABC, which may ultimately lead to personalized PABC treatment. This starts with clear insights in the histopathologic profile of PABC, which may identify clues for further in-depth research. However, the relative rarity of the disease precludes conducting large studies with sufficient patient numbers. Therefore, this study assesses the histopathological profile of PABC in a large Dutch population-based cohort, compared to age-matched non-pregnant breast cancer patients, to increase the understanding of the histopathologic characteristics of PABC. This will serve as a starting point for further in-depth molecular research within the same patient cohort.

## Methods

### Data source

Data were extracted from the nationwide Dutch network and registry of histo- and cytopathology (PALGA) [23], which contains excerpts of all pathology reports from Dutch laboratories [24]. All data within the PALGA research database are pseudonymized, both in the laboratories and by a trusted third party (ZorgTTP, Houten, The Netherlands). This study was approved by the PALGA scientific and privacy committee, and all data were handled in compliance with the General Data Protection Regulation Act (GDPR).

### Study population

Excerpts were extracted from all resection-specimen reports of women with a diagnosis of invasive breast cancer (IBC) and a mention of pregnancy, offspring, placenta, lactation, or abortion in their pathology report between January 1, 1988 and July 1, 2019 (*n* = 1941) (Supplementary Fig. 1). All patients with a diagnosis of IBC during pregnancy, or up to 6 months postpartum, were included, irrespective of pregnancy outcome, and type and timing of breast cancer treatment. Patients with sarcomas or a Phyllodes tumor, as well as patients with a history of breast cancer before their pregnancy, defined as a recurrence of invasive breast cancer in the contralateral or ipsilateral breast or chest wall at any time, were excluded (Supplementary Fig. 1).

A control cohort was drawn from unselected Dutch breast cancer patients (extracted from the same PALGA database) with a synoptic IBC resection-specimen report between January 1, 2013 and December 31, 2016 (*n* = 46,563 reports) (Supplementary Fig. 1). From this cohort, we excluded patients without a primary tumor in their resection specimen (*n* = 2104 reports) and patients ≥ 46 years old, as the eldest PABC patient was 45 years of age (*n* = 41,458 reports). In addition, males (*n* = 13) and overlapping (i.e., PABC) patients (*n* = 55) were excluded from this control cohort. Lastly, for patients with multiple reports, only the first report was included to prevent matching the same patient twice (Supplementary Fig. 1).

For both cohorts, we extracted clinicopathologic characteristics from the pathology reports, including histologic subtype, histologic grade, ER status, PR and human epidermal growth factor receptor 2 (HER2) status. For both cohorts, no information was available for the mode of presentation. However, presumably most patients were presented with a palpable mass, as no major screening program exists in the Netherlands for population below the age of 50 (except for patients known to have a hereditary predisposition). In addition, based on the receptor status for ER, PR, and HER2, tumors were sub-classified as ER/PR-driven (ER and/or PR+ , HER2), triple positive (TPBC: ER+ , PR+ , HER2+), triple negative (TNBC: ER−, PR−, HER2−), and HER2-driven (ER−, PR−, HER2+). Additionally, for the PABC cohort, gestational age at diagnosis was extracted, which was subdivided into the trimesters (trimester one: weeks 1–12, trimester two: weeks 13–26, trimester three: weeks 27–42), and the postpartum period (up to 6 months after delivery). For postpartum patients, a distinction was made between lactating and non-lactating women.

### Statistical analysis

Patients from both cohorts were randomly matched 1:1 on age. Clinicopathologic characteristics were summarized and differences between both cohorts were tested by means of a *χ*^2^ test for categorical variables. For the normally distributed continuous variable (age), a *t* test was performed. All tests were two-sided and *p*-values < 0.05 were considered statistically significant. All statistical analyses were performed using IBC SPSS statistics version 25.0.0.2.

## Results

### Patient characteristics

In total, 741 of 744 eligible patients with PABC could be age-matched to 741 non-PABC patients from the control cohort (Supplementary Fig. 1). Three patients have remained unmatched as there was no patient with similar age characteristics remaining in the control group to match against. Clinicopathologic variables of both cohorts are listed in Table 1.

**Table 1:** Characteristics of patients with pregnancy-associated breast cancer (PABC) (*n* = 741), age-matched 1:1 to non-PABC patients with invasive breast cancer (*n* = 741)

	PABC patients (*n* = 741)	Non-PABC patients (*n* = 741)	*p-*value
Age, median (range)	34.0 (19–45)^a^	34.0 (19–45)	1.000
Histological subtype, *n* (%)			
Ductal	707 (95.4%)	670 (90.4%)	0.000
Lobular	22 (3.0%)	31 (4.3%)	
Other	12 (1.6%)	40 (5.4%)	
Histological grade, *n* (%)			
Grade I	11 (1.5%)	92 (12.4%)	0.000
Grade II	124 (16.9%)	232 (31.3%)	
Grade III	595 (80.3%)	293 (39.5%)	
Unknown	11 (1.5%)	124 (16.7%)	
ER-receptor status, *n* (%)			
Positive	288 (38.9%)	505 (68.2%)	0.000
Negative	393 (53.0%)	210 (28.3%)	
Unknown	60 (8.1%)	26 (3.5%)	
PR-receptor status, *n* (%)			
Positive	251 (33.9%)	437 (59.0%)	0.000
Negative	415 (56.0%)	277 (37.4%)	
Unknown	75 (10.1%)	27 (3.6%)	
Her2-receptor status, *n* (%)			
Positive	149 (20.0%)	141 (10.0%)	0.000
Negative	483 (65.2%)	560 (75.6%)	
Unknown	109 (14.7%)	40 (5.4%)	
Gestational age			
First trimester	179 (24.2%)	N.a	
Second trimester	111 (15.0%)	N.a	
Third trimester	260 (35.1%)	N.a	
Postpartum: not lactating	94 (12.7%)	N.a	
Postpartum: lactating	83 (11.2%)	N.a	
Unknown gestational age	14 (1.9%)	N.a	

^a^Up to 6 months postpartum

The median age of patients in both cohorts was 34.3 years (range 19–45 years). Statistically significant differences (*p* < 0.0001) were observed for all other histopathologic characteristics. PABC patients had more often tumors of ductal type and higher grade (grade I: 1.5% vs. 12.4%, grade II: 16.9% vs. 31.3% and grade III: 80.3% vs. 39.5%) and these tumors were less often ER-receptor positive (38.9% vs. 68.2%) and PR-receptor positive (33.9% vs. 59.0%), as compared to the non-PABC breast cancer patients. In addition, tumors of PABC patients were more often HER2-receptor positive (20.0% vs. 10.0%). Surrogate intrinsic subtypes of patients with pregnancy-associated breast cancer are listed in Table 2.

**Table 2:** Surrogate intrinsic subtypes of patients with pregnancy-associated breast cancer (PABC) (*n* = 741), age-matched 1:1 to non-PABC patients with invasive breast cancer (*n* = 741)

	PABC patients (*n* = 741)	Non-PABC patients (*n* = 741)	*p-*value
Surrogate intrinsic subtypes, *n* (%)			
Triple positive			
Triple positive (ER+ , PR+ , Her2+)	63 (8.5%)	89 (12.0%)	0.000
Triple positive unknown (ER, PR, or HER2 missing)	112 (15.1%)	44 (5.9%)	
Triple negative			
Triple negative (ER−, PR−, Her2−)	284 (38.3%)	163 (22.0%)	0.000
Triple negative unknown (ER, PR, or HER2 missing)	112 (15.1%)	44 (5.9%)	
Hormone-driven			
Hormone-driven (ER and/or PR+ , Her2±)	281 (37.9%)	499 (67.3%)	0.000
Hormone-driven unknown (ER, PR, or HER2 missing)	112 (15.1%)	44 (5.9%)	
HER2-driven			
Her2-driven (ER± , PR± , Her2+)	149 (20.1%)	141 (19.0%)	0.000
HER2-driven unknown (ER, PR, or HER2 missing)	112 (15.1%)	44 (5.9%)	

### Intrinsic subtypes

Regarding the surrogate intrinsic subtypes, notable differences were observed for triple-negative and ER/PR-driven breast cancer. Breast tumors of PABC patients were significantly more often triple negative (38.3% vs. 22.0%, *p* < 0.0001) and significantly less often hormone-driven (37.9% vs. 67.3%, *p* < 0.0001).

### Gestational trimesters

The majority of PABC patients were diagnosed during pregnancy (74.2%), of which nearly half during the third trimester (47.3%) (Table 1). Of all pregnant PABC patients, 38 patients (5%) terminated their pregnancy in the first or second gestational trimester. Of the postpartum PABC patients, 83 patients were lactating (47%) and 94 patients (53%) did not breastfeed after pregnancy.

## Discussion

This large nationwide study compared the histopathologic profile of 741 PABC patients to an age-matched cohort of 741 non-pregnant breast cancer patients. A particularly aggressive histopathologic profile was observed for PABC patients, as their tumors were significantly more often of higher histologic grade, HER2 positive, and ER and PR negative. Furthermore, a higher incidence of triple-negative tumors in PABC patients was observed.

Our results are in line with previous studies in (smaller) PABC cohorts [10, 14, 15], which report comparable proportions of ER- and PR-negative tumors in PABC patients between 50 and 60%. In addition, higher grade tumors were also previously observed in PABC and patients [15]. Interestingly, the majority of our PABC patients were diagnosed during pregnancy, which is in contrast to a previous literature, claiming that two-thirds of patients are diagnosed postpartum (up to 12 months after delivery) [16, 25]. However, this may be due to our search strategy that depends on signs of pregnancy or the postpartum stage in pathology reports and inclusion of women with a diagnosis of invasive breast cancer during pregnancy or within 6 months postpartum. Perhaps a concurrent pregnancy is more likely to be mentioned in a pathology report than the postpartum stage (lactating or non-lactating). Our findings in this large nationwide cohort of PABC patients add to the existing literature, implicating that PABC may be a different breast cancer entity than breast cancer in non-pregnant young women. Although a more aggressive histopathologic profile is observed in young breast cancer patients in general [16, 26–28], PABC patients show an even more aggressive histopathologic profile. These findings render interesting clues for further studies to unravel the molecular and genetic background of PABC. For example, the high proportion of ER- and PR-negative tumors in PABC patients seems contradictory, as estrogen and progesterone levels are generally high during pregnancy. These tumors are therefore probably driven by other growth factors, or as recently suggested by Gupta et al., tumorigenesis may be driven by the influence of hormones on the host stroma, rather than the mammary epithelium itself [29]. This is supported by findings that xenograft models of PABC require systemic estrogen for their formation, and increasing the estrogen levels promotes the initiation and progression of ER-negative cancer (i.e., the tumor cells do not express estrogen receptors themselves) [29]. Further, PABC xenografts are rich in stroma and PABC cell lines do not proliferate in vitro (i.e., without their cancer-associated fibroblasts in response to estrogen). Furthermore, epigenetic changes, that by itself may affect subsequent hormone concentrations, could also play a role. In addition, a Norwegian study showed that lactating PABC patients have a worse outcome [30], which may indicate a prominent role for prolactin levels.

A limitation of this study is that data are drawn from pathology reports, which usually does not include clinical information, i.e., TNM stage, imaging, maternal treatment and neonatal and maternal outcome. This precludes outcome analyses like disease-free survival (DFS) and overall survival (OS) analyses. As a second step, these pathology data will therefore be linked to clinical and follow-up data from the Dutch Cancer Registry (NCR) to investigate whether the observed aggressive histopathologic profile translates into a worse survival for PABC patients. Another limitation of this study is the missing data, especially for HER2-receptor status in the PABC cohort. This is mostly due to the fact that routine HER2-testing was only introduced around the year 2000. However, it is unlikely that the distribution of HER2-receptor status in the population that has not received HER2-testing would differ from that was observed in patient who did receive HER2-testing.

There could be concerns about the time span in the different breast cancer cohorts: the PABC group covers a longer time period (1988–2019) in comparison with the non-PABC group (2013–2016). However, a short subgroup analysis between the PABC patients in three different time lines: 1988–2012, 2013–2016, and 2017–2019 observed no significant differences in receptor status or grade. Beside, histologic typing, grading, and detection of the ER and PR receptor by immunohistochemistry using monoclonal antibodies have not been changed in the last decades [31–33].

Overall, this large study nonetheless renders a unique, nationwide, overview of the histopathologic profile about all Dutch PABC patients since 1988. The observations from this study serve as a starting point for further in-depth research that may ultimately lead to tailored PABC treatment. Tumor tissue of al PABC patients is currently being collected from the concerning pathology laboratories. RNA- and DNA-sequencing will be performed. Further, the role of *BRCA1* and *BRCA2* germline mutations and prolactin levels will be investigated.

In conclusion, this large population-based cohort shows a significantly higher proportion of high-grade, and ER- and PR-negative tumors among PABC patients compared to age-matched controls. This underlines a different, more aggressive histopathologic profile for PABC. Further in-depth research will be conducted to unravel the genetic background of PABC, which may render clues for personalized PABC treatment.

## Supplementary Information

Below is the link to the electronic supplementary material.

Supplementary file1 (DOCX 103 kb)
