# Supplementary material for: Pregnancy-associated breast cancer: nationwide Dutch study confirms a discriminatory aggressive histopathologic profile
Source: Breast Cancer Res Treat. 2021 Feb 26;186(3):699–704. doi: 10.1007/s10549-021-06130-w (PMC8019425; doi:10.1007/s10549-021-06130-w)
Supplement: Supplementary file 1 — Supplementary file1 (DOCX 103 kb) [file 10549_2021_6130_MOESM1_ESM.docx]

**Supplementary figure 1.**

**Supplementary figure 1.** Flowchart of included patients with pregnancy-associated breast cancer (PABC) and breast cancer patients without PABC from the PALGA database.
